# Role and Function of MicroRNAs in Extracellular Vesicles in Cardiovascular Biology

**DOI:** 10.1155/2015/161393

**Published:** 2015-10-08

**Authors:** Philipp Pfeifer, Nikos Werner, Felix Jansen

**Affiliations:** Department of Internal Medicine II, University Hospital Bonn, Rheinische Friedrich-Wilhelms University, 53105 Bonn, Germany

## Abstract

Intercellular communication mediated by extracellular vesicles is crucial for preserving vascular integrity and in the development of cardiovascular disease. Extracellular vesicles consist of apoptotic bodies, microvesicles, and exosomes that can be found in almost every fluid compartment of the body like blood, saliva, and urine. In the recent years, a lot of reports came up suggesting that major cardiovascular and metabolic pathologies like atherogenesis, heart failure, or diabetes are highly influenced by transfer of microRNAs via extracellular vesicles leading to altered protein expression and phenotypes of recipient cells. The following review will summarize the fast developing field of intercellular signaling in cardiovascular biology by microRNA-containing extracellular vesicles.

## 1. Introduction

*Extracellular Vesicles.* Intercellular communication is essential for the maintenance of tissue homeostasis and disease development. Long known mechanisms of intercellular communication include direct cell-cell contact or the transfer of secreted molecules. In the last two decades, a third mechanism for intercellular communication has emerged that involves intercellular transfer of extracellular vesicles (EVs) [1].

EVs are small membrane vesicles, which are released by most cell types in the extracellular space. By containing and transferring various bioactive molecules to target cells, like proteins, RNAs, or microRNA (miR), EVs affect molecular pathways and biological behavior of recipient cells. EVs are heterogeneous in size and are released from cells under physiological and pathological conditions [2]. According to current definitions, EVs consist of three subgroups: exosomes (20–100 nm), microvesicles ((MVs) 0.1 *μ*m–1 *μ*m), and apoptotic bodies (0.5 *μ*m–2 *μ*m). Whereas exosomes are released continuously from cells, MVs and apoptotic bodies are predominantly liberated from activated or apoptotic cells [2, 3]. Exosomes are released from cells via the endolysosomal pathway. In contrast, MVs and apoptotic bodies are formed by budding from the plasma membrane. Therefore, the membrane composition of MVs or apoptotic bodies reflects that of the parent cell more closely than does the membrane composition of exosomes.

In 2007, it was first shown that EVs contain and transfer genetic information, in form of mRNAs and miRs, between mast cells regulating protein expression of recipient cells [4]. The notion that EVs might function as vector to transfer genetic information being able to regulate gene expression in target cell opened up a completely new field of research regarding intercellular communication mechanisms. Today, there is increasing evidence that the effect of EVs on target cells is mainly dependent on their intravesicular miR expression [5, 6]. By transferring miRs to target cells, EVs are now established as a novel layer in intercellular gene regulation [7].

*microRNA.* miRs are small, noncoding RNAs that provide posttranscriptional regulation of gene expression and control many (patho)physiological processes in cardiovascular health and disease [8]. miRs are short (18–25 nucleotides) noncoding RNAs transcribed in the nucleus and cleaved by the RNAse III enzyme Drosha to precursor hairpin miR (pre-miR). After transportation into the cytoplasm, the pre-miR is further processed into 18–25 nucleotide mature miR duplexes. Mature miRs can be loaded by the enzyme Dicer into the RNA-induced silencing complex (RISC), where protein expression of specific mRNA targets can be prevented by mainly two mechanisms. At sites with broad pairing complementarity, miRs can induce Argonaute-catalyzed mRNA cleavage [9]. More commonly, miRs direct translational repression, mRNA destabilization, or a combination of both including inhibition of translation initiation and poly(A) shortening [10]. Importantly, miRs not only exert their function intracellularly, but also can be exported from cells in the extracellular space via EVs or bound to proteins like Ago-2 or HDL [11–13]. In contrast to RNA, extracellular miRs show a high stability in fluids and are reliably detectable in the blood [14]. Therefore, miRs have emerged as a novel class of biomarkers for many diseases, such as cardiovascular disease [15]. The dual function of miRs as active effector of gene expression on one hand and as stable biomarker on the other hand opens up fascinating opportunities to improve the understanding and diagnosis of cardiovascular diseases. Of note, increasing evidence suggests that the stability of miRs in fluids is mediated by the protection of miRs from circulating RNAses through EVs [13]. Depending on the condition of the releasing cells, miR-incorporating EVs have been shown to regulate a multitude of divers functions in target cells mediating the maintenance of cardiovascular hemostasis or inducing cardiovascular pathologies, which will be the focus of the next paragraphs.

**Figure 1:**
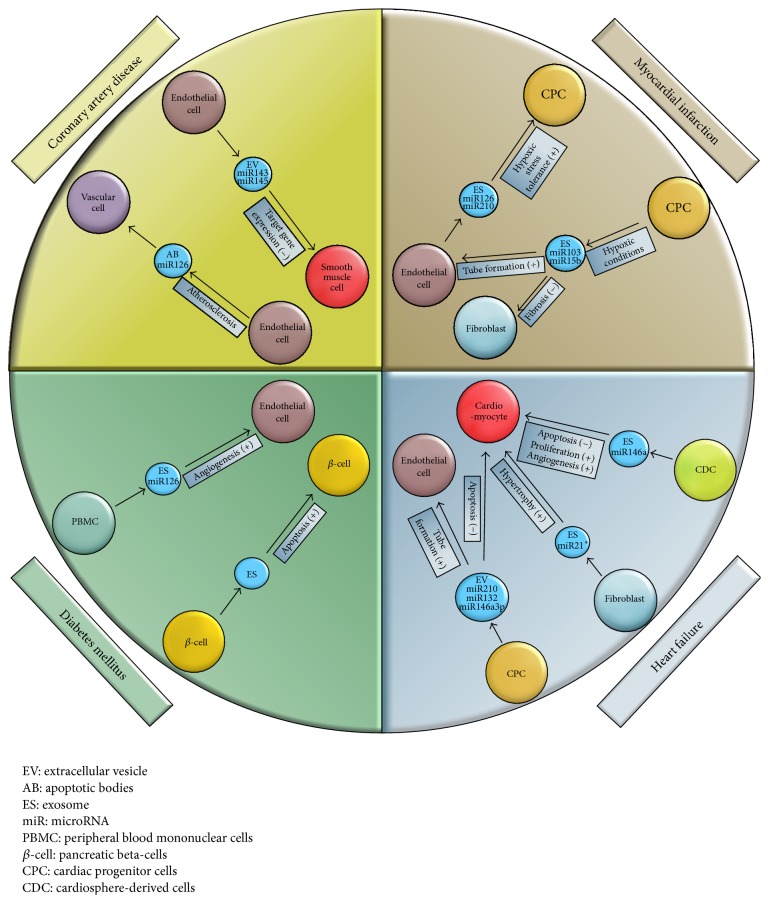
Intercellular signaling mechanisms via EV-bound microRNAs.

*EV microRNAs in Intercellular Communication.* See Figure 1. 

## 2. EV microRNAs: The Maintenance of Cardiovascular Homeostasis

EVs containing miRs are shed from a variety of cells under physiologic and pathophysiologic conditions. Considering the fact that most cellular mechanisms like cell growth, differentiation, apoptosis, and proliferation are regulated by miRs shows that they orchestrate the maintenance of cardiovascular homeostasis and thereby are promising targets for development of new therapeutical approaches [16]. There are several reports about beneficial biological effects of EVs-bound miRs. EVs from endothelial cells reduced atherosclerotic lesion formation in the aorta of ApoE^−/−^ mice and limited atherogenesis, improved plaque stability, and promoted incorporation of progenitor cells [17]. EVs from cardiac progenitor cells (CPC) inhibited apoptosis in mice-cardiomyocytes and injection of these CPC-EVs into infarcted hearts led to reduced cardiomyocyte apoptosis and improved LV ejection fraction [18]. Exosomes from CPCs are reported to improve cardiac function in a rat ischemia-reperfusion injury model and exosomes from endothelial cells increase tolerance to hypoxic stress in recipient CPCs (Figure 1) [18, 19]. Intravascular injection of endothelial MVs containing miR-126 accelerated reendothelialization after electric denudation of the endothelium in vivo [20]. Taken together, these findings illustrate the fundamental relevance of miR-delivering by EVs for preserving physiologic conditions in the body and emphasize the relevance of further investigations in this field aiming at a broadened understanding of their functions and development of new personalized drugs in the future.

## 3. Vascular Diseases

### 3.1. Coronary Artery Disease

Coronary artery disease (CAD) is the most common cause of death in the industrialized world [21]. In the last 5 years, many studies provided insights into the important role of miRs in the development of atherosclerosis by influencing inflammatory state, proliferation, and regeneration of vascular cells. miRs transfer by EVs contributes to maintenance of arterial homeostasis on the one hand but leads to inflammatory state in the interplay of endothelial cells (EC), smooth muscle cells (SMC), and immune cells on the other hand [16]. In the last years, a lot of reports have been published that show a close relationship between development of atherosclerosis and altered miR expression. For example, a recent publication showed that ApoE promotes the increase of miR-146a expression and thereby reduced NF-*κ*B signaling in monocytes and macrophages. In vivo, it could be pointed out that intravascular application of miR-146a mimetics leads to reduced atherogenesis [22]. Meiler et al. gained additional interesting results about the successful reduction of atherosclerotic plaque size. They proved that miR-302a regulates the expression of ATP-binding cassette (ABC) transporter ABCA1 mRNA and protein in primary macrophages. Moreover, they found that in vivo application of an anti-miR-302a in a low-density lipoprotein receptor deficiency (Ldlr^−/−^) mouse model leads to an increase in ABCA1 in the aorta as well as an increase in circulating plasma high-density lipoprotein levels by 35%. The anti-miR-302a-treated mice also displayed reduced atherosclerotic plaque size by 25% and a more stable plaque morphology with reduced signs of inflammation [23]. Zhang et al. examined that expression of miR-26a is reduced in the aortic intima of ApoE^−/−^ mice and treatment of human aortic endothelial cells with oxidized low-density lipoprotein (ox-LDL) suppressed miR-26a expression. In contrast, overexpression of miR-26a inhibited endothelial apoptosis and overexpression of TRPC6, a target of miR-26a, abolished the antiapoptotic effect of miR-26a [24]. A recent publication of our working group revealed that EMPs promote anti-inflammatory effects in vitro and in vivo by reducing endothelial ICAM-1 expression via the transfer of functional miR-222 into recipient cells and that EMP-mediated miR-222-dependent anti-inflammatory effects are reduced in pathological hyperglycaemic conditions [25]. Another study showed that exposing ECs to high glucose activates transcription of miR-503. Furthermore, it points out that miR-503 is packed into EMPs and delivered to vascular pericytes, resulting in reduced expression of EFNB2 and VEGFA in these cells, followed by impaired migration and proliferation [26]. Another publication discussing the involvement of miRs in the development of atherosclerosis was given by Di Gregoli et al. By investigating human coronary atherosclerotic plaques, they observed that increased matrix metalloproteinases- (MMP-) 14 protein expression in foam cell macrophages was associated with lesions exhibiting histological characteristics associated with an unstable phenotype. Added to that, they examined that microRNA-24 expression in these atherosclerotic plaques was inversely related to MMP-14 protein expression, that stable plaques contained higher microRNA-24 levels than unstable plaques, and that microRNA-24 colocalized with foam cell macrophages exhibited low MMP-14 protein expression. In ApoE^−/−^ mice, they found that microRNA-24 inhibition leads to increased plaque size and macrophage MMP-14 expression [27]. Schober et al. were able to observe that administration of miR-126-5p prevents atherosclerotic lesion formation by Notch1 inhibitor delta-like 1 homolog (Dlk1) suppression in an ApoE^−/−^ mouse model [28]. Sun et al. expanded our knowledge about influences of miRs on atherosclerotic development by several major findings. Firstly, they showed that miR-181b expression is reduced in the aortic intima and plasma in apolipoprotein E-deficient mice. Secondly, they determined that circulating miR-181b is markedly reduced in the plasma of human subjects with coronary artery disease. Moreover, they could show that systemic delivery of miR-181b suppressed NF-*κ*B signaling, reduced target gene expression in the aortic arch in apolipoprotein E-deficient mice, and significantly inhibited atherosclerotic lesion formation, proinflammatory gene expression, and the influx of lesional macrophages and CD4+ T cells in the vessel wall [29]. Hergenreider et al. found that Krüppel-like factor 2 (KLF2), a shear-responsive transcription factor, leads to upregulation of the miR-143/145 cluster in EC, while it is known that miR-143/145 controls SMC-phenotypes. They used KLF2-transduced or shear stress-stimulated human umbilical vein endothelial cells (HUVEC) to obtain miR­143/145 enriched EVs. Coculturing of HUVECs with SMCs induced reduced target gene expression in recipient SMCs (Figure 1). Furthermore, the authors could show that in an in vivo ApoE^−/−^ mice model EVs derived from KLF2-expressing EC reduced atherosclerotic lesion formation in the aorta [17]. Zernecke et al. gave another example for participation of EVs in development of atherosclerotic lesions. It is known that in response to tissue damage, the CXC chemokine CXCL12 and its receptor CXCR4 counteract apoptosis and recruit progenitor cells. On this basis they found that EC during atherosclerosis shed apoptotic bodies containing miR-126, leading to production of CXCL12 in recipient vascular cells (Figure 1). They examined miR-126 to repress the function of regulator of G protein signaling 16, an inhibitor of G protein-coupled receptor (GPCR) signaling, as underlying mechanisms. This leads to a feedback loop initiated by CXCR4, a GPCR, resulting in an increased production of CXCL12. Furthermore, they detected that treatment with apoptotic bodies, isolated from EC limited atherosclerosis, improved plaque stability and promoted the incorporation of Sca-1+ progenitor cells in a mouse model of atherosclerosis in an miR-126-dependent manner [30]. Another example for miR-participation in cellular processes involved in CAD development was given by Climent et al. They proved that SMCs transfer miR-143 and miR-145 to ECs, thereby modulating angiogenesis by reducing the proliferation of ECs and targeting hexokinase II (HKII) and integrin *β* 8 (ITG*β*8). In vivo studies showed that transforming growth factor (TGF) *β* and vessel stress initiated miR-143/145 transfer from SMCs to ECs by nanotubes [31]. Exploring the prognostic value of EV-bound miRs in a clinical study, our group pointed out that increased expression of miR-126 and miR-199a in circulating MVs was associated with a significantly lower major adverse CV event rate. EC and platelets were discovered as the major sources by generating an expression profile of miRs in MVs of 181 patients with stable coronary artery disease. In an experimental setting, we found that vascular endothelial repair is promoted by delivery of miR-126 containing endothelial microvesicles (EMV) and that these effects are altered under hyperglycaemic conditions, as occurring in diabetic patients [20, 32]. Another interesting mechanism was reported by Rautou et al., who observed that MVs isolated from human atherosclerotic plaques contain ICAM-1 and transfer this adhesion molecule to EC-membrane. ECs with increased ICAM-1 in the cell membrane in turn showed increased endothelial monocyte adhesion in cell culture and in isolated perfused mouse carotid. This mechanism probably contributes to atherosclerotic plaque progression [33]. In the last few years, many publications have been published about miR-regulation in endothelial cells caused by shear stress. Wang et al. showed that endothelial cells exposed to pulsatile shear stress for 24 h show altered miR-expression profiles. They demonstrated that the miR-23b cluster (miR-23b and miR-27b) is upregulated by pulsatile stress, whereas the miRs of miR-17–92 cluster (miR-17, miR-19b, miR-20a, miR-20b, and miR-92a), miR-16 cluster (miR-15b and miR-16), and miR-221 cluster (miR-221 and miR-222) are downregulated [34]. Another report revealed that shear stress leads to increased miR-27a/b expression in endothelial cells and that overexpression of miR-27a and miR-27b leads to increased endothelial cell sprouting [35]. Another report showed that miR-712 is upregulated by disturbed flow in endothelial cells, resulting in proatherogenic responses, endothelial inflammation, and permeability. Furthermore, they could demonstrate that miR-712 silencing by an anti-miR-712 prevents atherosclerosis in murine models of atherosclerosis [36]. Vion et al. identified shear stress exposition of endothelial cells as a regulator of microparticle release. They observed that low shear stress stimulates EMP release by activation of ERK1/2 pathways and Rho kinases, whereas high shear stress limits EMP release by regulating ABCA1 in a NO-dependent expression and by cytoskeletal reorganization [37]. There are several reports about shear stress-induced regulation of leukocyte adhesion to endothelial cells. Endothelial cells exposed to oscillatory shear stress show increased miR-21 and augmented VCAM-1 expression accompanied with higher adhesiveness of THP-1 cells, which could be attenuated by anti-miR-21 [38]. Endothelial cells exposed to low shear stress show increased inflammation caused by upregulation of miR-92a expression. Besides, overexpression of miR-92a leads to lower NO synthesis and enhanced monocyte adhesion to the endothelium [39–41]. Moreover, several publications show influences of shear stress on endothelial miR expression. Regarding miR-10a, it is reported that shear stress leads to increased expression of miR-10a resulting in inhibited NF-*κ*B activation and reduced endothelial inflammation [42]. miR-21 upregulation in endothelial cells can be achieved by exposing them to oscillatory shear stress (OSS). Upregulation of miR-21 results in reduced peroxisome proliferator-activated receptor (PPAR) *α*-translation, causing endothelial inflammation by inducing VCAM-1 and CCL2 expression [43]. Endothelial cells exposed to disturbed flow show miR-712 upregulation, promoting endothelial inflammation, and higher permeability of the endothelial barrier [36]. Interestingly, influences on atherosclerotic development by shear stress-induced miRs have been recently reported. For example, one report points out that expression of miR-19a is upregulated under shear stress exposition, resulting in cell-cycle arrest at G1-phase by cyclin D1 targeting [44]. Overexpression of miR-145 in SMCs leads to higher plaque stability and reduced plaque size in an in vivo mouse model [45]. Endothelial cells exposed to low shear stress combined with atherogenic oxLDL show upregulation of miR-92a expression. Application of an miR-92a antagomir in a ApoE^−/−^-mouse model leads to decreased atherosclerosis in antagomir-treated group compared to controls. Furthermore, atherosclerotic plaques of antagomir-treated mice showed higher collagen content, suggesting that lower expression of mir-92a leads to development of more stable plaques [39]. In a clinical study, it has been examined that miR-21 plasma levels of patients with diabetes type 2 are reduced compared to controls [46]. In circulating angiogenic progenitor cells (APCs) of patients with CAD, increased miR-21 levels could be observed compared to controls [47]. In vivo studies were able to point out that miR-10a is upregulated in endothelial cells from athero-protected sites of the aorta, while in endothelial cells from athero-prone sites of the aorta miR-21, miR-92a, mir-103, and miR-221 are upregulated [42, 48]. In the last years, several articles reported that miRs are involved in endothelial dysfunction. Transfection of endothelial cells with miR-221 and miR-222 reduced cell migration and overexpression of miR-221 and miR-222 resulted in reduced eNOS expression in endothelial cells [49, 50]. Xu et al. performed miRNA expression analysis of serum samples from atherosclerotic CAD patients. They observed that atherosclerotic CAD patients have increased expression levels of miR-135b-5p and miR-499a-3p in serum. Additionally, they identified miR-135b-5p and miR-499a-3p to repress myocyte enhancer factor 2C (MEF2C), leading to enhanced EC and VSMC proliferation and migration [51]. Cheng et al. activated endothelial cells by stimulating them with the proinflammatory cytokines IL-1*β* and TNF-*α* and observed upregulation of miR-146a and miR-146b. Next, they overexpressed miR-146a in endothelial cells and observed decreased expression of the inflammatory genes of VCAM-1, ICAM-1, SELE, and MCP-1 and an increased expression of eNOS mRNA, leading to decreased leukocyte adhesion. The authors concluded that miR-146a promotes decreased endothelial activation. Moreover, they observed that miR-146 negatively regulates proinflammatory NF-*κ*B-, MAP kinase-pathway, and downstream EGR transcription factors. Finally, they report that HuR, an RNA binding protein that promotes endothelial activation by suppressing expression of endothelial nitric oxide synthase (eNOS), is an miR-146 target [52]. In one publication of our own working group we generated EMP from HCAEC exposed to high glucose concentrations, defined as “injured” EMP (iEMP). It could be detected that iEMP injection significantly impaired endothelial function in ApoE^−/−^ mice, leading to increased macrophage infiltration and adhesion protein expression in atherosclerotic lesions of iEMP-treated ApoE^−/−^ mice by phosphorylation of p38 into its biologically active form phospho-p38 [53]. All these publications indicate the importance of miRs in maintaining vascular homeostasis on the one hand, but on the other hand they demonstrate that dysregulation of miRs in cardiovascular diseases gives rise to the development of atherosclerotic lesions. Further investigations are required to expand the knowledge about influences of miRs on vascular cell function and their role in the progression of atherosclerotic lesions in order to place the basis for development of new drugs and treatments of atherosclerosis.

### 3.2. Myocardial Infarction

In myocardial infarction (MI), a sudden thrombotic occlusion of a coronary vessel causes reduced oxygen supply to myocardial cells resulting in cell death. There are some studies elucidating the role of miRs and EVs in development of MI. A study of Gray et al. showed that CPCs secrete proregenerative exosomes under hypoxic conditions enhancing tube formation of ECs and decreasing profibrotic gene expression in TGF-*β*-stimulated fibroblasts. Microarray analysis of exosomes secreted by hypoxic CPCs identified 11 miRNAs that were upregulated compared with exosomes secreted by CPCs grown under normoxic conditions. Treatment of ECs and fibroblasts with exosomes from hypoxic CPCs revealed elevated miR-103 and miR-15b levels in recipient cells. Next, exosomes were used after ischemia-reperfusion injury in a rat model. The exosomes from hypoxic CPCs improved cardiac function, reflected by improved fractional shortening and reduced fibrosis (Figure 1) [18]. These findings indicate that hypoxia triggers a regenerative response by the delivery of exosomes from cardiac progenitor cells transferring antifibrotic miRs to fibroblasts. These findings were fostered by Ong et al., who examined that EC-derived exosomes can in turn be taken up by CPCs promoting antiapoptotic effects by transferring miR-126 and miR-210. They used a mouse model to prove that intramyocardial codelivery of a nonviral minicircle plasmid carrying HIF1 (MC-HIF1) together with CPCs leads to better survival of CPCs, when given after MI. Additionally, codelivery of MC-HIF1 with CPCs leads to improved echocardiographic ejection fraction. In vitro experiments revealed that EC produced exosomes that were internalized by recipient CPCs and that these exosomes overexpressing HIF1 had elevated contents of miR-126 and miR-210. These miRs activated prosurvival kinases and induced a glycolytic switch in recipient CPCs, providing them with increased tolerance to hypoxic stress in vitro. The inhibition of both of these miRs blocked the protective effects of the exosomes (Figure 1) [19]. Another very interesting report about the positive effects transduced by EVs was published by Lai et al. They treated mice after myocardial ischemia/reperfusion injury with exosomes gathered from medium of mesenchymal stem cells (MSC), leading to reduced infarct size [54]. Boulanger et al. were able to expand the knowledge about effects of MVs under MI condition by treating rat aortic rings with endothelium, using MV isolated from patients with acute MI. They observed that endothelium-dependent relaxation by acetylcholine was impaired in endothelium of aortic rings treated with MVs from patients with MI by impairing the endothelial nitric oxide transduction pathway [55]. Summarized, these reports show that EVs play a fundamental role in the reorganization of the heart muscle after MI and thus imply an enormous potential of interventional possibilities by admitting or decreasing miR-containing EVs.

## 4. Heart Diseases

### 4.1. Heart Failure

Heart failure (HF) is the result of cardiac remodeling caused by stress through a lot of adverse conditions, like CAD, atrial fibrillation, elevated blood pressure, and valvular heart disease. Cardiac remodeling leads to hypertrophy of cardiomyocytes and fibrosis of the heart muscle. In 2006, one study investigated increased expression of miRs in two mouse models (thoracic aortic banding (TAB); Tg mice expressing activated calcineurin A (can)) for cardiac hypertrophy and in hearts collected from patients with HF. Increased expression of miR-23, miR-24, mir-125b, miR-195, miR-199a, and miR-214 could be observed in hearts from patients with HF. Next, the authors used adenoviral vectors to overexpress these miRs in cultured myocytes. Overexpression of miR-23a, miR-23b, miR-24, miR-195, and miR-214 in cultured myocytes caused hypertrophic growth in vitro. In an in vivo mouse model overexpression of miR-195 promoted cardiac hypertrophy [56]. An intercellular communication mechanism between cardiac fibroblasts and cardiomyocytes was first shown by Bang et al. They revealed that miR transfer by exosomes plays an important role in the development of hypertrophy of cardiomyocytes. For example, they found that cardiac fibroblasts secrete exosomes containing miR passenger strands (“star” miRNAs), which are normally degraded intracellularly. Further investigations pointed out that miR-21_3p (miR-21^*∗*^), the passenger strand of miR-21, induces cardiomyocyte hypertrophy by targeting sorbin, SH3 domain-containing protein 2 (SORBS2), and PDZ and LIM domain 5 (PDLIM5). Furthermore, pharmacological inhibition of miR-21^*∗*^ reduced the development of cardiac hypertrophy in a Ang II mouse model. Taken together, this study could prove that fibroblast-derived exosomes enriched with miR-21^*∗*^ act as paracrine signaling mediator of cardiomyocyte hypertrophy (Figure 1) [57]. Another study showed that 16K PRL, a 16 kDa N-terminal prolactin fragment, induces miR-146a expression in ECs, leading to reduced angiogenesis by downregulation of NRAS and release of miR-146a-loaded exosomes. Absorption of these exosomes by cardiomyocytes resulted in decreased expression of Notch1, Erbb4, and Irak1. In a mouse model for peripartum cardiomyopathy (PPCM) with a cardiomyocyte-restricted Stat3 knockout (CKO mice) elevated cardiac miR-146a expression and simultaneously downregulated of* Erbb4*,* Nras*,* Notch1*, and* Irak1* were found, which could be attenuated when miR-146a was blocked with locked nucleic acids or antago-miR. Measurement of miR-146a in hearts and plasma levels of PPCM patients revealed elevated miR-146a levels [58]. In conclusion, this study presents miR-146a-loaded exosomes shed by ECs as important messengers in the development of PPCM, which can be attenuated by antago-miRs. Furthermore, it delivers evidence that miR-146a-loaded exosomes could play an important role as biomarker for diagnosis of PPCM in the future. Barile et al. described that medium from CPCs inhibited apoptosis in cardiomyocytic mouse cells and enhanced tube formation in HUVECs by EVs containing miR-210, miR-132, and miR-146a-3p (Figure 1). miR-210 downregulated ephrin A3 and PTP1b, which caused inhibition of apoptosis in cardiomyocytic cells, whereas miR-132 inhibited RasGAP-p120, resulting in increased tube formation in ECs. Besides, they demonstrated that injection of EVs from CPCs into infarcted hearts was associated with less cardiomyocyte apoptosis, enhanced angiogenesis, and improved LV ejection fraction compared with a control group [59]. Also, cardiosphere-derived cells (CDCs) are involved in exosome secretion, which causes antiapoptotic and enhanced proliferative and angiogenic effects on cardiomyocytes. For that reason, exosomes were injected in injured mouse hearts and could recapitulate the regenerative effects of CDC transplantation, while blocking of exosome production by CDCs attenuated these effects. Exosomes of CDCs showed high levels of miR-146a and administration of an miR-146a mimic showed some benefits of CDC exosomes (Figure 1) [60]. In conclusion, the presented studies display the important role of miR transfer by EVs in the development of heart failure and the protection against undesired modifications of the heart muscle. Further investigations are necessary for a more detailed understanding of the effects of EVs and to enable the development of new extracellular vesicle based therapies in the future.

### 4.2. Cardiac Hypertrophy

Several reports show that miRs play a functional role in cardiac hypertrophy. Roncarati et al. created a miR profile of 41 hypertrophic cardiomyopathy (HCM) patients. HCM patients were diagnosed by transthoracic echocardiography and cardiac magnetic resonance and healthy people, who were matched by age and sex, were used as control.

In plasma of HCM patients 12 miRs were significantly increased, but only miR-29a was significantly associated with hypertrophy and fibrosis. The authors concluded that miR-29a is a potential biomarker for HCM assessment [61]. Further reports about generating miR-expression profiles from patients with cardiac diseases can be found. For instance, Nair et al. analyzed miR-expression profile of three different kinds of patients with dilated cardiomyopathy (DCM): patients with DCM and isolated diastolic dysfunction, patients with stable compensated DCM, and patients with decompensated congestive heart failure secondary to DCM (DCM-CHF). The expression profile showed that mir-142-3p was decreased in patients with DCM and DCM- CHF, while miR 124-5p was increased in patients with DCM [62]. A similar report compared expression profile of miRs in patients with Takotsubo cardiomyopathy, healthy individuals, and ST-segment elevation acute myocardial infarction (STEMI) patients. They examined that patients with Takotsubo cardiomyopathy had special miR-1, miR-16, miR-26a, and miR-133a profile compared with the other groups, which could be used as a biomarker to distinguish Takotsubo cardiomyopathy patients from STEMI patients [63].

The presented studies are of particular interest, as the planning of an adequate therapy strategy in treatment of diseases is of great importance to enable the separation of low- and high-risk patients. Due to their different underlying therapy strategies, the necessity increases to distinguish between MI and Takotsubo cardiomyopathy in clinical daily life.

## 5. Diabetes Mellitus

Diabetes mellitus (DM) is a metabolic disorder characterized by dysfunction of insulin-secreting pancreatic beta-cells with great importance for the development of CAD. It is well known that miRs regulate beta-cell activity, but recently some studies reported that these miRs are also transferred from beta-cells to other recipient cells via exosomes. For instance, cytokine-treated MIN6B1 cells secrete exosomes containing miRs that are transferred to neighboring beta-cells, leading to apoptosis. MIN6B1 cells were treated with cytokines (IFN*γ*, TNF-*α*, and IL-1*β*) and exosomes were isolated from the culture media. Giving these exosomes to MIN6B1 or mice islet cells leads to apoptosis in recipient cells (Figure 1). Furthermore, they showed that miRs released in MIN6B1 exosomes do not simply reflect the content of the cells of origin, but a subset of miRs was preferentially released in exosomes, while others were selectively retained in the cells. Interestingly, exposition of MIN6B1 cells to inflammatory cytokines changed the release of several miRs [64]. This study gave new insights into the contribution of exosomes to the vanishing of insulin-secreting pancreatic beta-cells and thereby include the possibility to develop new therapies on this pathomechanism. Additional knowledge about mechanisms taking place in diabetic conditions was gained by Barutta et al. They reported that urinary exosomes from patients with microalbuminuria contain increased concentrations of miR-130a and miR-145, while the amount of miR-155 and miR-424 is reduced. In an animal model of early experimental diabetic nephropathy urinary exosomal miR-145 levels were increased while simultaneously miR-145 within the glomeruli was overexpressed. In addition, cultured mesangial cells exposed to high glucose showed increased miR-145 content in mesangial cells and their associated exosomes [65]. In 2010, Zampetaki et al. generated an expression profile of miRs in plasma of patients with DM. They observed lower plasma levels of miR-20b, miR-21, miR-24, miR-15a, miR-126, miR-191, miR-197, miR-223, miR-320, and miR-486 in prevalent DM, but a modest increase of miR-28-3p. Added to that, they were able to point out a reduction of miR-126 content in endothelial apoptotic bodies under high glucose concentrations [46]. The next study provides an interesting insight into the way miR-containing EMPs influence inflammatory effects in diabetic state. EMVs promote anti-inflammatory effects in vitro and in vivo by reducing endothelial ICAM-1 expression as they transfer functional miR-222 into recipient cells. Intriguingly, anti-inflammatory effects were reduced under hyperglycaemic conditions due to reduced miR-222 content of generated EMP [25]. Mocharla et al. were able to detect that CD34^+^ peripheral blood mononuclear cells (PBMC) shed higher levels of miR-126 containing exosomes than CD34- PBMC subsets and that the exosomes containing higher miR-126 levels had higher proangiogenic effects on ECs than lower miR-126 level containing exosomes (Figure 1). At the same time, they also reported that treatment of CD34^+^-PBMC with anti-miR-126 or inhibition of their release lowered their proangiogenic effects. Beyond that, they observed that treatment of CD34^+^ PBMC with high-glucose levels and growing CD34^+^ PBMC under diabetic conditions showed reduced miR-126 levels accompanied with impaired proangiogenic properties, which could be rescued by miR-mimic-126 treatment [66]. As the pancreatic rest function is often accompanied with the necessity of insulin injection in addition to oral therapy, the long-term conservation of pancreatic rest function in diabetic patients must be targeted. The presented studies offered new insights into mechanisms leading to apoptosis of insulin-secreting pancreatic beta-cells. Furthermore, new biomarkers for microalbuminuria were presented by a special miR-expression profile in the urine of patients with microalbuminuria, possibly helping to diagnose microalbuminuria in earlier stages in the future.

## 6. miR-Based Therapeutics

In general, there are two possible therapeutic approaches of using miRs. On the one hand, overexpressed miRs could be suppressed by application of miRs with a complementary sequence to the target miRs (anti-miRs) and, on the other hand, downregulated miRs with an appreciated effect could be precipitated by application of oligonucleotides mimicking endogenous miRs [67]. Recent reports have raised hope that targeting of miRs could serve as a new therapeutic approach. For example, one study reported that Miravirsen, a locked nucleic acid-modified DNA phosphorothioate antisense oligonucleotide, is able to suppress effects of miR-122, and by that it is able to reduce hepatitis C virus RNA [68]. Under hypoxic conditions from CPCs, generated exosomes improved cardiac function and reduced fibrosis in a rat ischemia-reperfusion injury model [18]. Fiedler et al. showed in a mouse model that antago-miRs were able to reduce endothelial apoptosis, enhance vascularization, decrease infarct size, and improve cardiac function after MI [69]. Kumarswamy et al. proved in a transaortic constriction (TAC) mouse model that miR-21 silencing antago-miRs were able to reduce cardiac dysfunction and fibrosis [70]. A mouse model of limb ischemia and MI indicated that intravenous application of an miR-92a antago-miR was able to induce enhanced angiogenesis and functional improvement of damaged tissue [71]. In vivo studies pointed out successful miR downregulation by adenovirus-transfer. Therefore, an arterial balloon injury in a rat model was combined with an adenovirus mediated transfer of miR-126-3p target sites, which was able to inhibit proliferation of vascular SMCs and to attenuate restenosis [72]. Taken together, there is a bunch of promising reports about positive effects of antago-miRs in a variety of diseases. But it should be concerned that a lot of obstacles have to be managed until miR- or antago-miR-based drugs will find broad way into the clinic.

### 6.1. Vesicle-Incorporated miRs as Novel Therapeutic Tool?

EVs containing miRs represent a promising new therapeutic approach, because of their important natural roles in cellular processes like proliferation, differentiation, and apoptosis combined with high stability, tissue-specific expression pattern, and secretion to body fluids [73]. Among all EVs, exosomes seem to be the most suitable vehicle for miR delivery, because they physiologically target specific cells due to the proteins contained in their membranes. This enables them to specifically bind to recipient cell receptors, providing the possibility to create exosomes that specifically target one desired cell type. This fact signifies a big step in the development of personalized medicine. Besides, they are flexible in cargo type, are nonimmunogenic, and maintain the cargo stable for delivery [73]. Meanwhile, there are several reports of exosomes used as therapeutic agents. For example, Lai et al. reported that mesenchymal stem cell (MSC) derived exosomes reduce infarct size in a mouse model of myocardial ischemia/reperfusion injury [54]. Added to that, it is reported that miR-150 is selectively packaged into MVs of monocytes and can be taken up by ECs leading to enhanced cell migration [74]. Besides therapeutic tools of miRs incorporated in extracellular vesicles, like exosomes, many reports using nanoparticles as a new approach to transport miRs to recipient cells have been published in the last few years. Cheng and Saltzman developed biodegradable polymer nanoparticles, which are coated with cell-penetrating peptides that can effectively deliver chemically modified oligonucleotide analogues to achieve gene regulation. This nanoparticle system could block the activity of the oncogenic miR-155, as well as attenuating the expression of the protooncogene, Mcl-1, leading to reduced cell viability and proapoptotic effects in the recipient cells [75]. Furthermore, Babar et al. inhibited miR-155 by delivery of antisense peptide nucleic acids encapsulated in polymer nanoparticles and thereby slowed down pre-B-cell tumors growth in vivo [76]. An integrin *α*v*β*3-targeted nanoparticle was used by Anand et al. to deliver anti-miR-132 to the tumor endothelium of human breast carcinoma in mice, causing restored p120RasGAP expression in the tumor endothelium and thereby suppressed angiogenesis and decreased tumor burden [77]. Chen et al. took a LPH (liposome-polycation-hyaluronic acid) nanoparticle formulation modified with tumor-targeting single-chain antibody fragment (scFv) for systemic delivery of miR-34a into lung metastasis of murine B16F10 melanoma, prompting significant downregulation of survivin expression in the metastatic tumor and reduced tumor load in the lung [78]. Su et al. reported that systemic delivery of a chemically stabilized anti-miR-122 complexed with interfering nanoparticles (iNOPs) effectively silences the liver-expressed miR-122 in mice. miR-122 is a liver-specific miRNA, with suggested roles in cholesterol, fatty acid, and lipid metabolism [79]. Although these articles focused on miR delivery using nanoparticles mainly as therapeutic tool to combat cancer, it is reasonable that nanoparticles can also be used to deliver miRs to recipient vascular cells for influencing inflammation and development of atherosclerosis.

### 6.2. Current Limitations and Future Perspective/Directions

Unfortunately, the usage of miRs in EVs as therapeutical approaches is limited by the amount of miRs that have to be transferred in order to have an effect on recipient cells. It could be shown that a lot of miRs did not show any detectable activity and that some miRs only had weak effect on mRNA silencing, because of an inappropriate target-to-miR ratio [80]. Besides this, the increase of their binding specificity, circulation time, and protection from cleavage by nucleases still needs to be managed. To handle these problems, miRs need chemical modifications, like locked nucleic acids (LNAs) or 2′′′′-O-methylation and have to be packed into vehicles, like lipids, vectors, or polymers [67]. In perspective, circulating miRNAs could revolutionize diagnosis and estimation of prognosis of cardiovascular diseases by providing new biomarkers. But it should also be considered that no possible biomarker has been validated in large cohort studies until now [81]. Despite these promising results, a lot of issues need to be addressed until all advantages of EV-bound miRs can be used in full range. It will still need a lot of investigation and time to develop EVs that do not have any off-target effects and any immunogenicity and whose long-term effects are known [73]. Moreover, it must be assumed that miR expression profile changes in different disease states and thereby determination of appropriate endogenous controls will be complicated. Apart from that, the detection of suitable clinical methods to quantify circulating miRNA, also associated with upcoming costs, must be put in focus [81].

## 7. Conclusions

EV-driven miR transfer in CAD is a new promising field of research giving new insights in protein regulation, pathomechanisms of diseases, and modulation of cellular phenotypes and will possibly deliver new biomarkers and therapeutical approaches in the future.
